# Supplementary figures and images for: Random Matrix Analysis for Gene Interaction Networks in Cancer Cells
Source: Sci Rep. 2018 Jul 13;8:10607. doi: 10.1038/s41598-018-28954-1 (PMC6045654; doi:10.1038/s41598-018-28954-1)

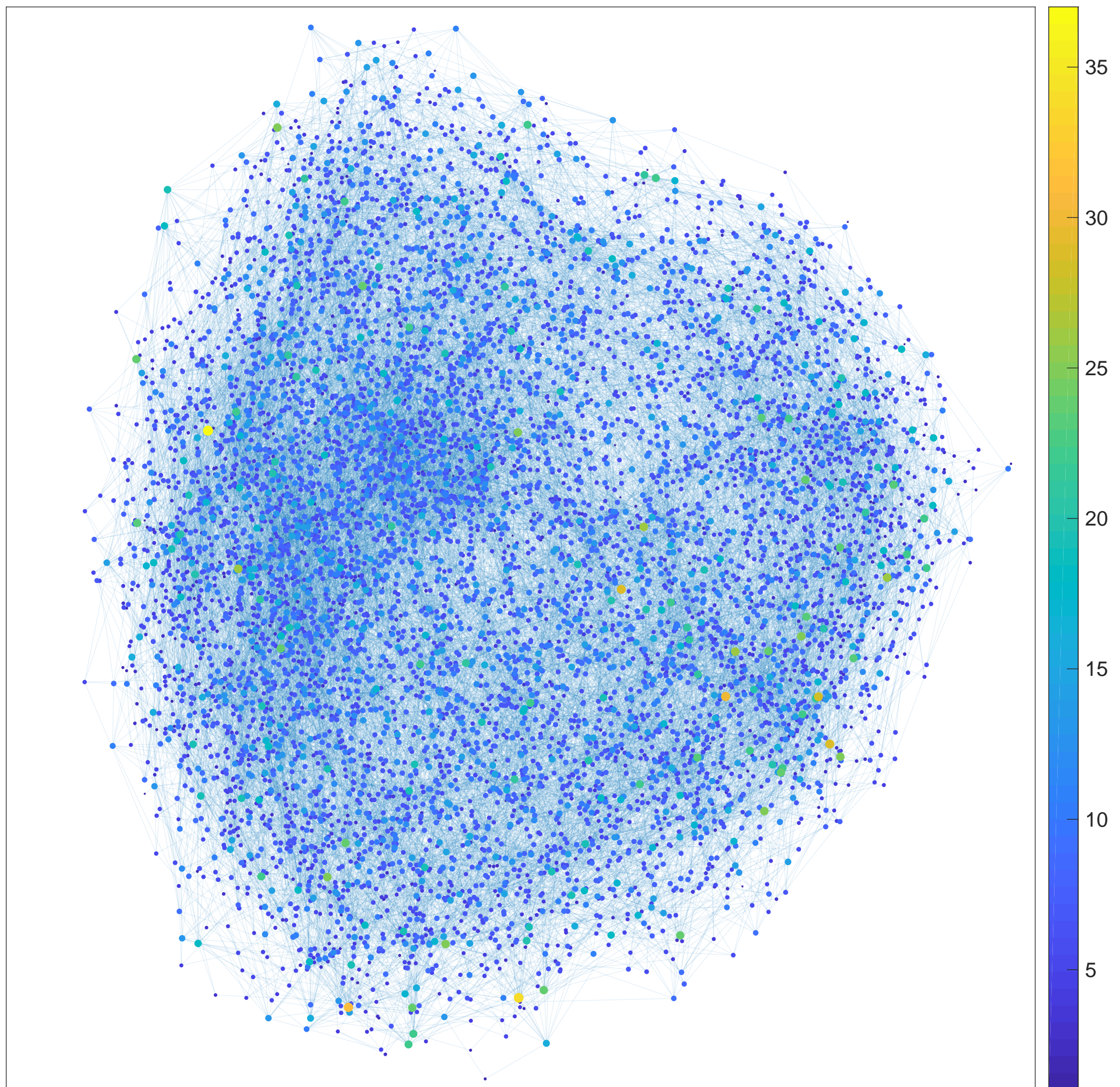

Supplement: Supplementary file 3 — Supplementary Figure S1 [file 41598_2018_28954_MOESM3_ESM.pdf]

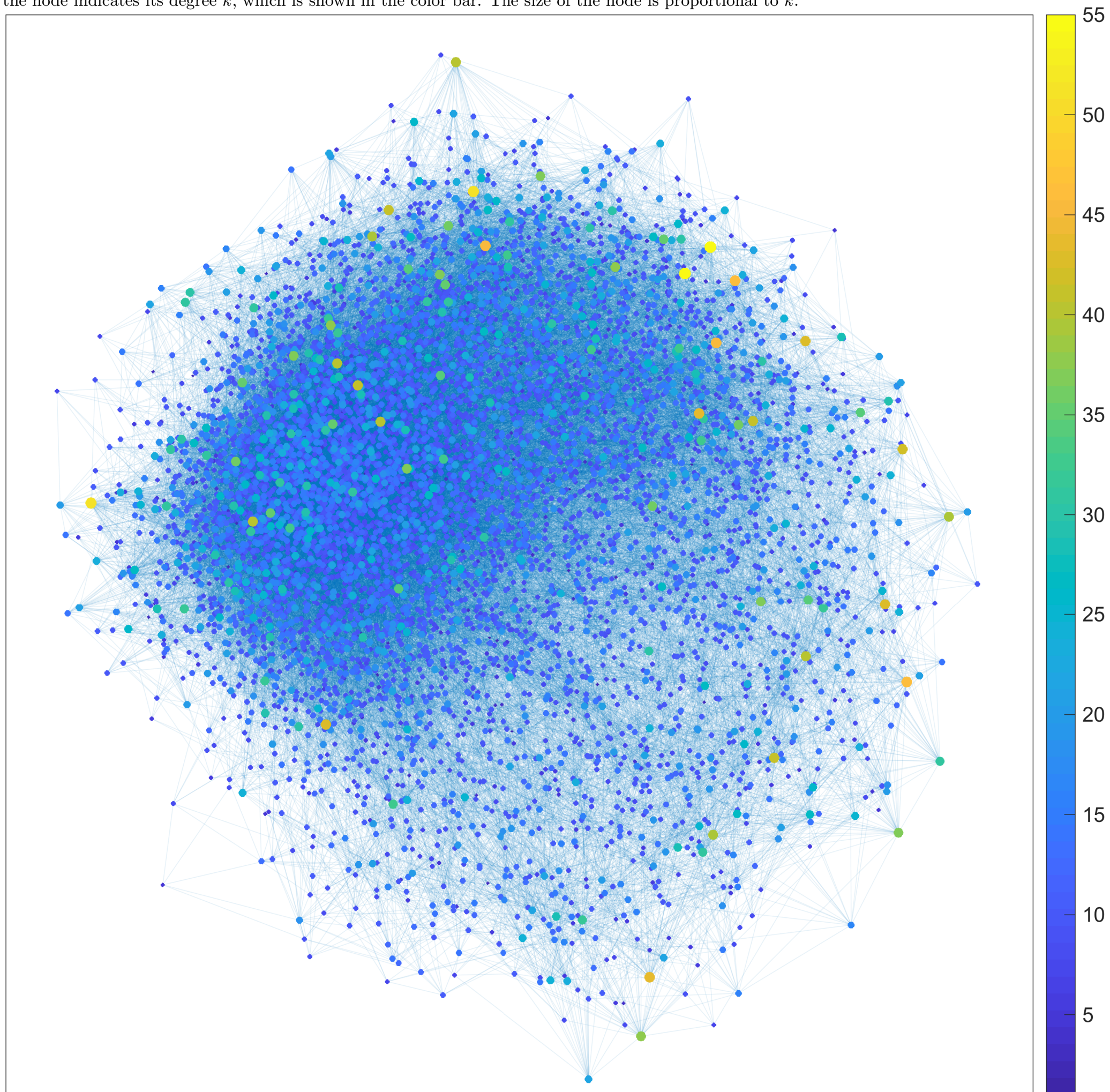

Supplement: Supplementary file 4 — Supplementary Figure S2 [file 41598_2018_28954_MOESM4_ESM.pdf]

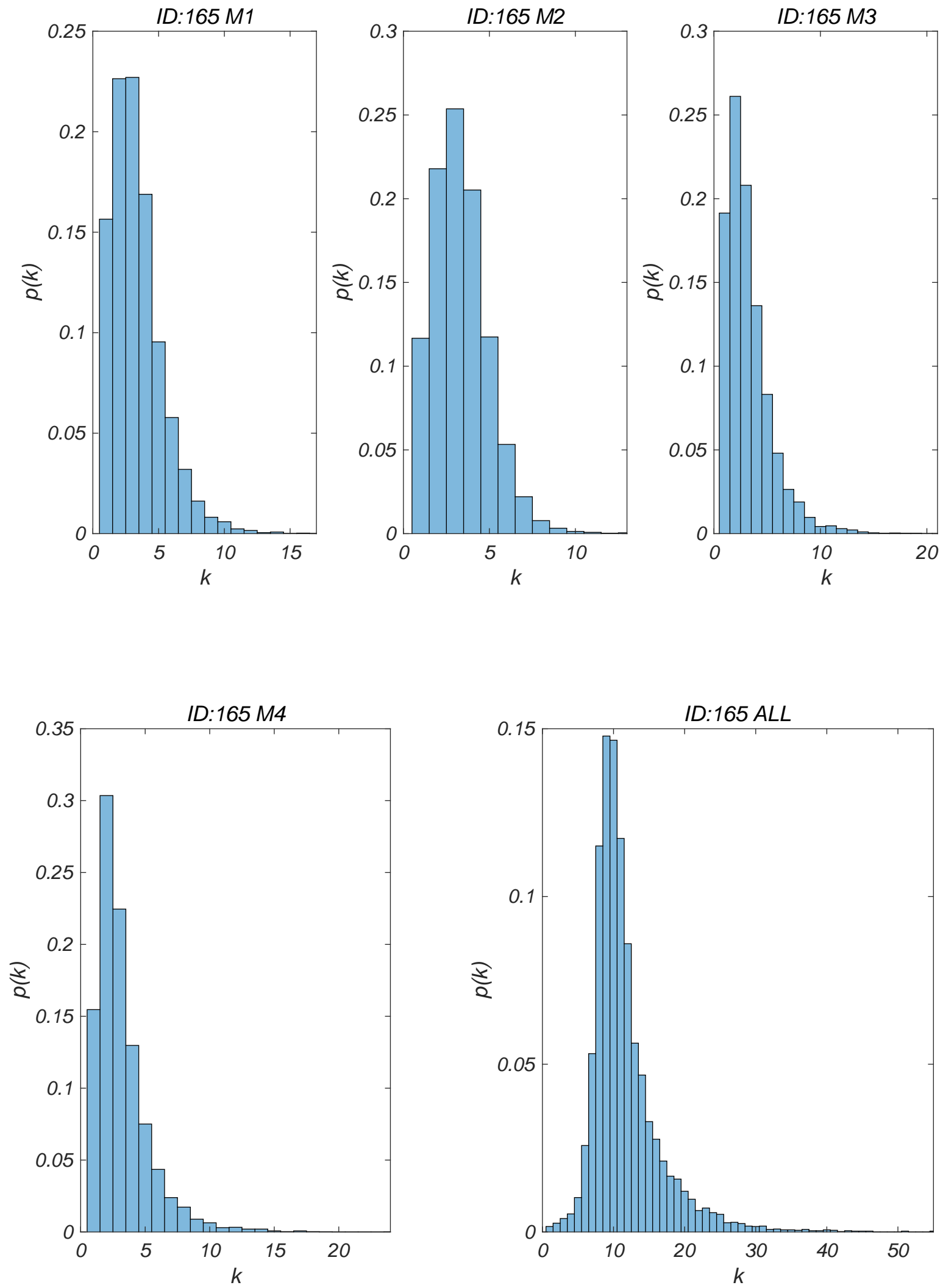

Supplement: Supplementary file 6 — Supplementary Figure S4 [file 41598_2018_28954_MOESM6_ESM.pdf]
