# Supplementary material for: Random Matrix Analysis for Gene Interaction Networks in Cancer Cells
Source: Sci Rep. 2018 Jul 13;8:10607. doi: 10.1038/s41598-018-28954-1 (PMC6045654; doi:10.1038/s41598-018-28954-1)

RANDOM MATRIX ANALYSIS FOR GENE INTERACTION NETWORKS IN CANCER CELLS, Ayumi Kikkawa

Supplementary Figure S3 : The probability distribution of the node degree $p(k)$.

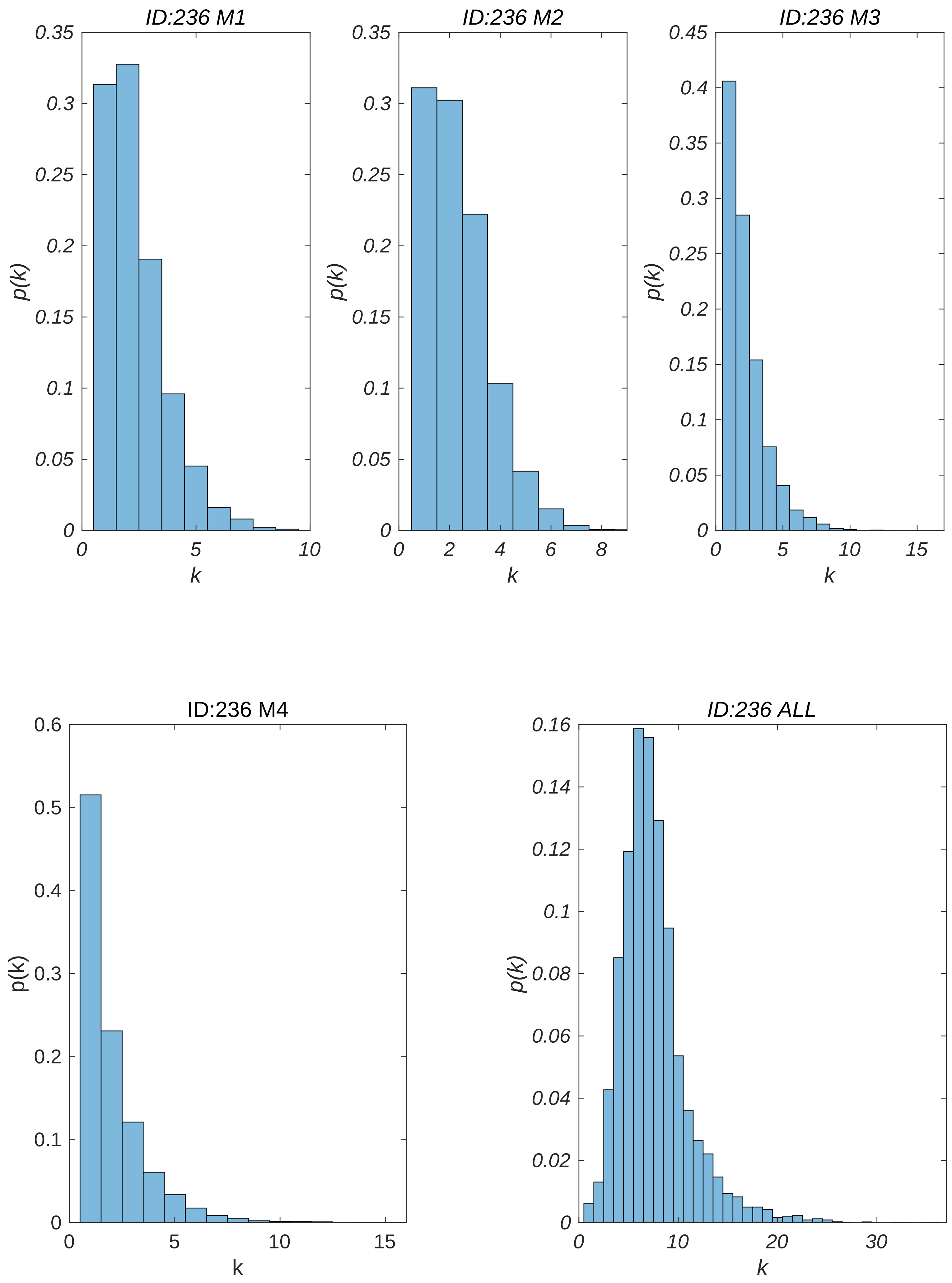

Supplement: Supplementary file 5 — Supplementary Figure S3 [file 41598_2018_28954_MOESM5_ESM.pdf]
